# Note on fractional Mellin transform and applications

**DOI:** 10.1186/s40064-016-1711-x

**Published:** 2016-02-01

**Authors:** Adem Kılıçman, Maryam Omran

**Affiliations:** Department of Mathematics, Institute for Mathematical Research, University Putra Malaysia, 43400 Serdang, Selangor Malaysia; Institute for Mathematical Research (INSPEM), University Putra Malaysia, 43400 Serdang, Malaysia

**Keywords:** Fractional calculus, Riemann–Liouville fractional integral and derivative, Caputo fractional derivative, Mellin transform

## Abstract

In this article, we define the fractional Mellin transform by using Riemann–Liouville fractional integral operator and Caputo fractional derivative of order $\alpha \ge 0$ and study some of their properties. Further, some properties are extended to fractional way for Mellin transform.

## Background

Mellin transform occurs in many areas of engineering and applied mathematics. According to Flajolet et al. (1995), Hjalmar Mellin (1854–1933) gave his name to the Mellin transform that associates to a function *f*(*x*) defined over the positive reals, the complex function ${\mathcal{M}}[f(x);s]$. It is closely related to the Laplace and Fourier transforms. We start by recalling the definition and some important properties of the Mellin transform. The domain of definition is an open strip, $\langle a, b \rangle ,$ of complex numbers $s = \sigma + it$ such that $0 \le a < \sigma < b$. Here we recollect the definition from Flajolet et al. (1995) and some properties which are mentioned in Kiliçman (2006).

### **Definition 1**

(*Flajolet et al.*^1995^) Let *f*(*x*) be locally Lebesgue integrable over $(0, \infty )$. The Mellin transform of *f*(*x*) is defined by

$$M[f(x);s] = f^{*}(s)=\int _{0}^{\infty }{x^{s-1}f(x)dx}.$$

The largest open strip $\langle a, b \rangle$ in which the integral converges is called the fundamental strip. The inverse Mellin transform is defined as the following:

### **Theorem 1**

*Letf*(*x*) *be integrable with fundamental strip*$\langle \alpha ,\beta \rangle$. *Ifcis such that*$\alpha <c<\beta$*and*$f^{*}(s=c + it) ={\mathcal{M}}[f(x);s]$*is integrable, then the equality*

$$f(x)=\dfrac{1}{2\pi i}\int _{c-i\infty }^{c+i\infty } {\mathcal{M}}\left[ f(x);s \right] x^{-s}ds$$

*holds almost everywhere. Moreover, iff*(*x*) *is continuous, then equality holds everywhere on*$( 0, +\infty).$

### **Theorem 2**

(Kiliçman 2004) *Letfbe Mellin transformable function defined on*${\mathbb{R}}_{+}$. *If differentiation under the integral sign is allowed, then we have*

(1): $${\mathcal{M}}[f^{(n)}(x);s]= \int _{0}^{\infty }f^{n}(x)x^{s-1}dx=\dfrac{(-1)^{n}\Gamma (s)}{\Gamma (s-n)}M[f(x);s-n]$$
(2): $${\mathcal{M}}[x^{n}f^{(n)}(x);s]=\int _{0}^{\infty }x^{n}f^{n}(x)x^{s-1}dx=(-s)^{n}M[f(x);s]$$
(3): $$\left( \dfrac{d}{ds}\right) ^{n} {\mathcal{M}}[f(x);s]=\int _{0}^{\infty }f(x)(\log x)^{n}x^{s-1}dx=M[(\log x)^{n}f(x);s-1]$$
(4): $${\mathcal{M}}\left[ \int _{0}^{x}f(t)dt;s\right] =\int _{0}^{\infty }\left( \int _{0}^{x}f(t)dt\right) x^{s-1}dx=-\frac{1}{s}M[f(x);s+1].$$

*For more information readers may refer to Butzer and Jansche* (1997),* Erdélyi et al.* (1954),* Flajolet et al.* (1985),* Podlubny* (1999) *and  Butzer and Jansche* (1998), and (Kiliçman 2006).

## Basic definitions of fractional calculus

Fractional calculus is a generalization of the classical calculation and it has been used successfully in various fields of science and engineering. In fact, there are new opportunities in mathematics and theoretical physics appear, when order differential operator or operator becomes an integral arbitrary parameter. The fractional calculus is a powerful tool for the physical description systems that have long-term memory and long term spatial interactions see Podlubny (1999), Miller and Ross (1993), Hilfer (2000), Kilbas et al. (2006) and Samko et al. (1993).

There are different types of fractional derivatives in the current literature. One of the new fractional derivatives that was recently proposed is called Caputo–Fabrizio derivative see Atangana (2016), Caputo and Fabrizio (2015) and Losada and Nieto (2015). However in our study, Riemann–Liouville and Caputo derivatives have been used.

The use of integral transforms to deal with fractional derivatives traces back to Riemann and Liouville (Oldham and Spanier 1974; Widder 1971). Further, in Dattoli et al. (2003) the authors have shown that combined use of integral transforms and special polynomials provides a powerful tool to deal with fractional derivatives and integrals.

In this section, we give the definitions of Riemann–Liouville and Caputo fractional operators along the main properties as follows:

### **Definition 2**

A real function *f*(*x*), $x>0$ is said to be in space $C_{\mu } , \mu \in {\mathbb{R}}$ if there exists a real number $p>\mu$, such that $f(x)=x^{p}f_{1}(x)$ where $f(x)\in C(0,\infty )$, and it is said to be in the space $C_{\mu } ^{n}$ if $f^{n}\in C_{\mu } ,n\in {\mathbb{N}}.$

### **Definition 3**

The Riemann–Liouville fractional derivative operator of order $\alpha$ of a function *f*(*x*) is defined as:

1 $$D^{\alpha }f(x)=\frac{1}{\Gamma (m-\alpha )}\int _{0}^{x}(x-t)^{m-\alpha -1}f(t)dt, \quad m-1<\alpha <m.$$

### **Definition 4**

(*Podlubny*^1999^) The Riemann–Liouville fractional integral operator of order $\alpha \ge 0$ of a function $f\in C_{\mu }, \mu \ge -1$ is defined as:

2 $$D^{-\alpha }f(x)=J^{\alpha }f(x)=\frac{1}{\Gamma (\alpha )}\int _{0}^{x}(x-t)^{\alpha -1}f(t)dt, \quad t > 0, \quad \alpha >0$$

in particular $$J^{0}f(x)=f(x).$$

**Some properties of Riemann–Liouville fractional operator**

If $\alpha , \beta$ are two positive real number, then:

1. $$D^{\alpha }(D^{-\beta }f(x))=D^{\alpha -\beta }f(x),$$
2. $$J^{\alpha }J^{\beta }f(x)=J^{\alpha +\beta }f(x),$$
3. $$J^{\alpha }J^{\beta }f(x)=J^{\beta }J^{\alpha }f(x).$$

### **Definition 5**

(*Caputo*^1969^) The Caputo fractional derivative of $f\in C_{-1}^{m} , m\in {\mathbb{N}},$ is defined as

$$\begin{aligned} D_{c}^{\alpha }f(x)&= {} J^{m-\alpha }D^{n}f(x) \\ D_{c}^{\alpha }f(x)&= {} \frac{1}{\Gamma (m-\alpha )}\int _{0}^{x}(x-t)^{m-\alpha -1}f^{m}(t)dt,\quad m-1 <\alpha \le m, \end{aligned}$$

for $$m-1<\alpha \le m$$, $$m\in {\mathbb{N}}.$$

### **Theorem 3**

*If*$m-1 <\alpha \le m , m\in N , f\in C_{\mu }^{m}, \mu >-1,$*then the following two properties hold*

(1): $$D_{c}^{\alpha }\left[ J_{c}^{\alpha }f(x)\right] =f(x),$$
(2): $$J^{\alpha }\left[ D_{c}^{\alpha }f(x)\right] =f(x)-\sum\nolimits _{k=0}^{m-1}f^{k}(0)\left( \dfrac{x^{k}}{k!}\right).$$

### **Definition 6**

(*Fractional Cauchy’s integral formula*) (Jumarie 2010) Assume that $f: U\rightarrow {\mathbb{C}}$, $z \rightarrow f(z)$ is a fractional analytic function of order $\alpha = \frac{1}{{N}}, {N} \ge 1$, *N* integer. For every $a\in U$ consider the disk $D \subset U$ with the boundary defined by the circle $\gamma$ of which the radius is *r*. Then *f* (*z*) is actually infinitely $\alpha$th differentiable, with

$$f^{(n\alpha )}(a)=\dfrac{(n\alpha )!}{(2\pi i)^{\alpha }{N}^{\alpha }} \oint _{c(0,N)}\frac{f(z)}{(z-a)^{(n+1)\alpha }}dz ,\quad \alpha =\dfrac{1}{{N}}.$$

As a special case when $n=1,$ the fractional derivative can be written in the form:

$$f^{(\alpha )}(a)=\dfrac{(\alpha )!}{(2\pi i)^{\alpha }{N}^{\alpha }} \oint _{c(0,{N})}\dfrac{f(z)}{(z-a)^{2\alpha }}dz ,\quad \alpha =\dfrac{1}{{N}}.$$

## Main results

In this part, some properties of Mellin transform of fractional operator have shown.

### **Theorem 4**

*Letf*(*x*) *be Mellin transformable function on*$(0,\infty ),$*where*$0\le n-1< \alpha <n,$*then*

(1): $${\mathcal{M}}[D_{c}^{\alpha } J_{c}^{\alpha } f(x) ;s]={\mathcal{M}}[ f(x) ;s]$$,
(2): $${\mathcal{M}}[J^{\alpha }D_{c}^{\alpha } f(x) ;s]={\mathcal{M}}[ f(x);s ]-\sum\nolimits_{k=0}^{m-1} \dfrac{f^{k}(0)}{k!(k+s)}$$, $$\ Re(s)>-Re(k)$$,
(3): $${\mathcal{M}}[J^{\alpha }J^{\beta }f(x);s]=\dfrac{\Gamma (1-\alpha -\beta -s)}{\Gamma (1-s)} M[f(t);\alpha +\beta +s]$$.

### *Proof*

1. The result is obtained by applying Mellin transform to both sides of the first property (1) in Theorem 3
  $${\mathcal{M}}\left[ D_{c}^{\alpha }J_{c}^{\alpha }f(x);s\right] ={\mathcal{M}}[f(x);s].$$

(2) We apply Mellin transform on the part (2) in Theorem 3, then we obtain
  $$\begin{aligned} {\mathcal{M}}\left[ J^{\alpha }D_{c}^{\alpha }f(x);s\right]&= {} {\mathcal{M}}[f(x);s]-{\mathcal{M}}\left[ \sum\limits_{k=0}^{m-1}\dfrac{f^{k}(0)x^{k}}{k!};s\right] \\&= {} {\mathcal{M}}[f(x);s]-\sum\limits_{k=0}^{m-1}\dfrac{f^{k}(0)}{k!}\int _{0}^{\infty }x^{k+s-1}dx \\&= {} {\mathcal{M}}[f(x);s]-\sum\limits_{k=0}^{m-1}\dfrac{f^{k}(0)}{k!(k+s)},\quad Re(s)> -Re(k) \end{aligned}$$

(3) Now,we are applying Mellin transform of $J^{\alpha } J^{\beta }$
  $$\begin{aligned} {\mathcal{M}}\left[ J^{\alpha } J^{\beta } f(x);s\right] ={\mathcal{M}}\left[ J^{\alpha +\beta } f(x);s\right]&= {} \int _{0}^{\infty }x^{s-1}\frac{1}{\Gamma (\alpha +\beta )}\int _{0}^{x} (x-t)^{\alpha +\beta -1}f(t)dtdx \\&= {} \dfrac{1}{\Gamma (\alpha +\beta )}\int _{0}^{\infty }f(t)dt\int _{t}^{\infty }x^{s-1}(x-t)^{\alpha +\beta -1}dx. \end{aligned}$$
  Setting $x=\frac{t}{u}$, then the *x*-integral becomes
  $$t^{\alpha +\beta +s-1}\int _{0}^{1}u^{-\alpha -\beta -s}(1-u)^{\alpha +\beta -1}du.$$
  So,
  $$\begin{aligned} {\mathcal{M}}\left[ J^{\alpha } J^{\beta } f(x);s\right]&= {} \dfrac{1}{\Gamma (\alpha +\beta )}\int _{0}^{\infty }t^{\alpha +\beta +s-1}f(t)dt\int _{0}^{1}u^{-\alpha -\beta -s}(1-u)^{\alpha +\beta -1}du,\\&\quad \hbox{where}\quad Re(\alpha +\beta )> 0,\quad Re(\alpha +\beta +s)<1. \end{aligned}$$
  After using beta function which is defined by $B(\alpha ,\beta )=\int _{0}^{1}t^{\alpha -1}(1-t)^{\beta -1}dt$ and the fact that $B(\alpha ,\beta )=\dfrac{\Gamma (\alpha )\Gamma (\beta )}{\Gamma (\alpha +\beta )},$ hence obtain,
  $$\begin{aligned} {\mathcal{M}}\left[ J^{\alpha } J^{\beta } f(x);s\right]&= {} \frac{\Gamma (1-\alpha -\beta -s)}{\Gamma (1-s)}\int _{0}^{\infty }t^{\alpha +\beta +s-1}f(t)dt\\&= {} \frac{\Gamma (1-\alpha -\beta -s)}{\Gamma (1-s)} {\mathcal{M}}[f(t);\alpha +\beta +s]. \end{aligned}$$

### **Theorem 5**

*Letfbe Mellin transformable defined on*${\mathbb{R}}_{{+}}$, then

1. ${\mathcal{M}}\left[ f^{\frac{1}{2}}(x);s\right] =\int _{0}^{\infty }x^{s-1}f^{\frac{1}{2}}(x)dx ,$ by using fractional integration by parts and fractional derivative of power function, we obtain
  $$\begin{aligned} {\mathcal{M}}\left[ f^{\frac{1}{2}}(x);s\right]&= {} \int _{0}^{\infty }x^{s-1}f^{\frac{1}{2}}(x)dx =\int _{0}^{\infty }f(x)D^{\frac{1}{2}}x^{s-1}dx \\&= {} \dfrac{\Gamma (s)}{\Gamma (s-\frac{1}{2})}{\mathcal{M}}\left[ f(x);s-\frac{1}{2}\right] \end{aligned}$$

(2) ${\mathcal{M}}\left[ f^{\frac{3}{2}}(x);s\right] =\int _{0}^{\infty }x^{s-1}f^{\frac{3}{2}}(x)dx,$ by using fractional integration by parts and fractional derivative of power function, we obtain
  $$\begin{aligned} {\mathcal{M}}\left[ f^{\frac{3}{2}}(x);s\right]&= {} \int _{0}^{\infty }x^{s-1}f^{\frac{3}{2}}(x)dx =\int _{0}^{\infty }f(x)D^{\frac{3}{2}}x^{s-1}dx \\&= {} \dfrac{\Gamma (s)}{\Gamma (s-\frac{3}{2})}{\mathcal{M}}\left[ f(x);s-\frac{3}{2}\right]. \end{aligned}$$

Continuing by the induction, then the results in Theorem 5 can be extended to further fractional derivatives as the following theorem:

### **Theorem 6**

*Letfbe Mellin transformable function on*${\mathbb{R}}_{+}$, *andfis a fractional derivative function for all*$n-1<\alpha <n,\ n\in {\mathbb{N}}$, *then:*

$${\mathcal{M}}\left[ f^{\alpha }(x);s\right] =\dfrac{\Gamma (s)}{\Gamma (s-\alpha )}{\mathcal{M}}\left[ f(x);s-\alpha \right].$$

### *Remark 4*

By using the same technique in above theorem, Mellin transform of fractional integral can be yielded as the following formula:

$${\mathcal{M}}\left[ I^{\alpha }f(x);s\right] = \dfrac{\Gamma (s)}{\Gamma (s+\alpha )}{\mathcal{M}}\left[ f(x);s+\alpha \right].$$

### **Theorem 7**

*Letfbe Mellin transformable defined on*${\mathbb{R}}_{{+}}$, *then*

$$\begin{aligned} (1)\;{\mathcal{M}}\left[ x^{\frac{1}{2}}f^{\frac{1}{2}}(x);s\right] =\int _{0}^{\infty }f^{\frac{1}{2}}(x)x^{s-\frac{1}{2}}dx&= {} \dfrac{\Gamma (s+\frac{1}{2})}{\Gamma (s)}{\mathcal{M}}\left[ f(x);s \right]. \\ (2)\;{\mathcal{M}}\left[ x^{\frac{3}{2}}f^{\frac{3}{2}}(x);s\right] =\int _{0}^{\infty }f^{\frac{3}{2}}(x)x^{s+\frac{1}{2}}dx&= {} \dfrac{\Gamma (s+\frac{3}{2})}{\Gamma (s)}{\mathcal{M}}\left[ f(x);s \right]. \end{aligned}$$

By the same way as in Theorem 5, the next result follows:

$${\mathcal{M}}\left[ x^{\alpha }f^{\alpha }(x);s\right] =\dfrac{\Gamma (s+\alpha )}{\Gamma (s)}{\mathcal{M}}\left[ f(x);s \right].$$

### *Example 1*

Solve the problem:

$$x^{\frac{1}{2}}f^{\frac{1}{2}}(x)+x^{\frac{3}{2}}f^{\frac{3}{2}}(x)=\delta (x-a)$$

By applying the Mellin transform to both side and on using the Theorem 7 we have

$$\dfrac{\Gamma \left(s+\frac{1}{2}\right)}{\Gamma (s)}{\mathcal{M}}\left[ f(x);s \right] +\dfrac{\Gamma\left(s+\frac{3}{2}\right)}{\Gamma (s)}{\mathcal{M}}\left[ f(x);s \right] =a^{s-1}.$$

By solving the equation and applying the inverse Mellin transform by using complex inversion integral in order to cover the *f*(*x*) explicitly as the solution

$$f(x)=\dfrac{1}{2\pi i}\int _{c-i\infty }^{c+i\infty } \dfrac{\Gamma (s)}{\Gamma \left( s+\frac{1}{2}\right) +\Gamma \left( s+\frac{3}{2}\right) }a^{s-1}x^{-s}ds.$$

### **Theorem 8**

*Let*$f\in X_{(a,b)}$*and holomorphic on the stripSt*(*a*, *b*). *In additionfis Mellin transformable function, then*

3 $$\left( \frac{d}{ds}\right) ^{\alpha }{\mathcal{M}}[f(u);s]=\frac{1}{(1-\alpha )!}{\mathcal{M}}\left[ (\log u)^{\alpha }f(u);s\right],$$

*where*$$s\in St(a,b),$$*and*$$0\le \alpha \le 1$$.

### *Proof*

We set $\varphi (x)= s+\delta e^{ix}$, where *St*(*a*, *b*) contains the circle ${C}_{\delta }(s)$ of radius $\delta$ with *s*.

First of all, for $u > 0,$ let us consider

$$\begin{aligned} \left( \frac{d}{ds}\right) ^{\alpha } u^{s-1}&= {} \frac{1}{(1-\alpha )!}\left( u^{s-1}\right) ^{1-\alpha }\left( u^{s-1}\log u\right) ^{\alpha }\\&= {} \frac{1}{(1-\alpha )!} u^{s-1}(\log u)^{\alpha }. \end{aligned}$$

Secondly, we apply fractional Cauchy’s integral formula when $n=1$ for fractional derivatives

$$\begin{aligned} \frac{1}{(1-\alpha )!}u^{s-1}(\log u)^{\alpha }=\left( \frac{d}{ds}\right) ^{\alpha }u^{s-1}&= {} \frac{(\alpha )!}{(2\pi i)^{\alpha }N^{\alpha }} \oint _{C_{\delta }(s)}\frac{u^{z-1}}{(z-s)^{2\alpha }} dz\\&= {} \frac{(\alpha )!}{(2\pi i)^{\alpha }N^{\alpha }}\int _{0}^{2\pi }\frac{u^{\varphi (x)-1}}{(\varphi (x)-s)^{2\alpha }}\varphi ^{\prime }(x) dx\\ \int _{0}^{\infty }\frac{1}{(1-\alpha )!}u^{s-1}(\log u)^{\alpha }f(u)du&= {} \frac{(\alpha )!}{(2\pi i)^{\alpha }N^{\alpha }}\int _{0}^{2\pi }\frac{\varphi ^{\prime }(x)}{(\varphi (x)-s)^{2\alpha }} \int _{0}^{\infty } \left\{ u^{\varphi (x)-1}f(u)du\right\} dx\\ \frac{1}{(1-\alpha )!}{\mathcal{M}}[(\log u)^{\alpha }f(u);s]&= {} \frac{(\alpha )!}{(2\pi i)^{\alpha }N^{\alpha }}\int _{0}^{2\pi }\frac{{\mathcal{M}}[\varphi (x);s]\varphi ^{\prime }(x)}{(\varphi (x)-s)^{2\alpha }}dx \end{aligned}$$

By another application of fractional Cauchy’s integral formula, we obtain

$$\frac{1}{(1-\alpha )!} {\mathcal{M}}\left[ (\log u)^{\alpha }f(u);s\right] = \left( \frac{d}{ds}\right) ^{\alpha }{\mathcal{M}}[f(u);s]$$

Therefore, the proof of Theorem 8 is fulfilled. $\square$

### *Example 2*

Let $f(x)=e^{-x}$ we apply Theorem 8 then we have

$$\frac{1}{(1-\alpha )!} {\mathcal{M}}\left[ (\log x)^{\alpha }e^{-x};s\right] =\left( \frac{d}{ds}\right) ^{\alpha }{\mathcal{M}}\left[ e^{-x};s\right]= \left( \frac{d}{ds}\right) ^{\alpha }\Gamma (s).$$

So,

$${\mathcal{M}}\left[ (\log x)^{\alpha }e^{-x};s\right] =(1-\alpha )!\left( \frac{d}{ds}\right) ^{\alpha }\Gamma (s),\quad Re (s) >0.$$

### *Example 3*

Let *f*(*x*) be Delta function, $f(x) = \delta (x-a)$, $a>0$ and by Theorem 8, then we get

$$\begin{aligned} \frac{1}{(1-\alpha )!} {\mathcal{M}}\left[ (\log x)^{\alpha }\delta (x-a);s\right]&= {} \left( \frac{d}{ds}\right) ^{\alpha }{\mathcal{M}}[\delta (x-a);s]\\&= {} \left( \frac{d}{ds}\right) ^{\alpha }a^{s-1}=\frac{1}{(1-\alpha )!}a^{s-1}(\log a)^{\alpha } \end{aligned}$$

Thus, $${\mathcal{M}}\left[ (\log x)^{\alpha }\delta (x-a);s\right] =a^{s-1}(\log a)^{\alpha }.$$

### *Remark 5*

For special cases we have the following:

1. If $\alpha =1$ then the formula (3) turns to [part (3) in Theorem 2] when $n=1,$
2. In the Example 2,
  (i) if $$\alpha =0$$ then $${\mathcal{M}}[e^{-x};s]= \Gamma (s),$$
  (ii) if $\alpha =1$ then we get the result
      $${\mathcal{M}}\left[ (\log x)e^{-x};s\right] =\left( \frac{d}{ds}\right) \Gamma (s)\quad \hbox{where}\quad Re (s)>0 ,\quad \hbox{see Oberhettinger (1974)},$$
3. In the Example 3,
  (i) if $\alpha =0$ then we obtain ${\mathcal{M}}[\delta (x-a);s]=a^{s-1},$ see Oberhettinger (1974),
  (ii) if $\alpha =1$ then we obtain the same result in Graf (2010) when $n=1$
      $${\mathcal{M}}\left[ (\log x)\delta (x-a);s\right] =a^{s-1}(\log a),$$

### **Theorem 9**

*Let*${\mathcal{M}}[f(x);s]$*be Mellin transform of the functionf*(*x*) *in*$(0,\infty ),$*where*$0<3\alpha <1$, *then*

(1): $${\mathcal{M}}\left[ D^{3\alpha } f(x);s\right] = \dfrac{\Gamma (s)}{\Gamma (s)-3\alpha }{\mathcal{M}}[f(x);s-3\alpha ]$$,
(2): $${\mathcal{M}}\left[ D^{\alpha }D^{\alpha }D^{\alpha }f(x);s\right] =\dfrac{(\Gamma (s))^{3}}{(\Gamma (s-\alpha ))^{3}}{\mathcal{M}}[f(x);s-3\alpha ]$$.

### *Proof*

1. The result is given directly from Theorem 6 then we obtain
  $${\mathcal{M}}\left[ D^{3\alpha }f(x);s\right] =\dfrac{\Gamma (s)}{\Gamma (s)-3\alpha }{\mathcal{M}}\left[ f(x);s-3\alpha \right] ,\quad {\rm {where}} \quad 0 <3\alpha <1.$$

(2) Also we apply the formula in Theorem 6 part by part as the following:
  $$\begin{aligned} {\mathcal{M}}\left[ D^{\alpha }D^{\alpha }D^{\alpha }f(x);s\right]&= {} \dfrac{\Gamma (s)}{\Gamma (s-\alpha )}{\mathcal{M}}\left[ D^{\alpha }D^{\alpha }f(x);s-\alpha \right] \\&= {} \dfrac{\Gamma (s)}{\Gamma (s-\alpha )}\dfrac{\Gamma (s)}{\Gamma (s-\alpha )}{\mathcal{M}}\left[ D^{\alpha }f(x);s-2\alpha \right] \\&= {} \dfrac{\Gamma (s)}{\Gamma (s-\alpha )}\dfrac{\Gamma (s)}{\Gamma (s-\alpha )}\dfrac{\Gamma (s)}{\Gamma (s-\alpha )}{\mathcal{M}}\left[ f(x);s-3\alpha \right] \\&= {} \dfrac{(\Gamma (s))^{3}}{(\Gamma (s-\alpha ))^{3}}{\mathcal{M}}[f(x);s-3\alpha ]. \end{aligned}$$
  From (1) and (2) we observe that
  $${\mathcal{M}}\left[ D^{\alpha }D^{\alpha }D^{\alpha }f(x);s\right] \ne {\mathcal{M}}\left[ D^{3\alpha } f(x);s\right].$$

## Conclusion

In this paper, some properties of fractional calculus are proposed by applying Mellin integral transform, and some applications are also given. Further, the results in fractional sense by using Mellin transform are in agreement with ordinary way in the existing literature. In fact, Mellin integral transform and its inverse are powerful to solve some kinds of fractional equations with variable coefficients, that will be a future study.
